# Supplementary material for: Dihydrogen Bonding—Seen through the Eyes of Vibrational Spectroscopy
Source: Molecules. 2022 Dec 28;28(1):263. doi: 10.3390/molecules28010263 (PMC9822382; doi:10.3390/molecules28010263)
Supplement: Supplementary file 1 [file molecules-28-00263-s001.zip › molecules-2114038-supplementary.pdf]

Supporting Materials: Dihydrogen Bonding - Seen Through the Eyes of Vibrational Spectroscopy

Marek Freindorf, Margaret McCutcheon, Nassim Beiranvand, and Elfi Kraka*

Computational and Theoretical Chemistry Group (CATCO)
Department of Chemistry, Southern Methodist University
3215 Daniel Ave, Dallas, Texas 75275-0314, USA

Friday 16th December, 2022

Contents

1 NBO hydrogen charges for reference compounds 2

2 Dihydrogen bonded complexes investigated in our study 3

*email: ekraka@smu.edu

1 NBO hydrogen charges for reference compounds

Table S1: NBO hydrogen charges [a] for reference compounds.

	Molecule	$q(H^{\delta+})$	$q(H^{\delta-})$	Molecule	$q(H^{\delta+})$	$q(H^{\delta-})$
Monomers	LiH		-0.795	HOCl	0.580	
	BeH		-0.434	KrH		-0.221
	BH4		-0.073	ArF	0.258	
	CH4	0.206		KrF	0.104	
	NH3	0.352		C2H2	0.230	
	NH4	0.460		C2HF	0.240	
	H2O	0.460		HCN	0.225	
	H3O	0.580		HNC	0.438	
	HF	0.549		CHF	0.157	
	NaH		-0.800	CHF2	0.124	
	AlH3		-0.392	CHO	0.154	
	AlH4		-0.410	CFO	0.492	
	GaH3		-0.301	HNO	0.238	
	SiH4		-0.158	H2NO	0.420	
	HCl	0.263		BeBe		-0.458
	HOCl2	0.583		FeH	0.145	

[a] $q(H^{\delta+})$: protonic atom charge (e), $q(H^{\delta-})$: hydridic atom charge (e).

2 Dihydrogen bonded complexes investigated in our study

ωB97X-D/aug-cc-pVTZ level of theory, bond length values R in Å, force constant k^a in mdyn/Å, vibrational frequency ω_a in cm^{-1}, energy E in kcal/mol, electron density at critical points ρ_c in e/Å^3, energy density at critical points H_c in Hartree/Å^3.

Table S2: Dihydrogen Bonded Complexes, ωB97X-D/aug-cc-pVTZ level of theory[a].

Molecule	Bond	R	k^a	Δk^a	$\Delta k^a/k^a$	ω_a	$\Delta\omega_a$	$\Delta\omega_a/\omega_a$	E	ρ_c	H_c	H_c/ρ_c	n
LiC1	Li-H	1.614	0.978	0.001	0.001	1373	2	0.001		0.261	-0.003	-0.012	
	H-H	2.495	0.025			290			-0.73	0.037	0.003	0.088	0.191
	H-C	1.089	5.319	-0.037	-0.007	3116	-11	-0.004		1.925	-2.154	-1.119	
LiC2	Li-H	1.641	0.817	-0.160	-0.163	1255	-116	-0.085		0.246	-0.001	-0.005	
	H-H	2.502	0.034			336			-14.03	0.062	0.001	0.021	0.208
	H-C	1.089	5.298	0.029	0.006	3110	9	0.003		2.024	-2.293	-1.133	
LiC3	Li-H	1.640	0.822	-0.155	-0.158	1258	-113	-0.082		0.248	-0.002	-0.007	
	H-H	2.316	0.049			408			-12.42	0.079	0.001	0.011	0.230
	H-C	1.091	5.199	-0.058	-0.011	3081	-17	-0.005		2.087	-2.396	-1.148	
LiC4	Li-H	1.609	0.978	0.001	0.001	1373	2	0.001		0.259	0.000	-0.001	
	H-H	1.780	0.130			661			-8.83	0.137	-0.005	-0.036	0.301
	H-C	1.088	4.897	-1.389	-0.221	2990	-398	-0.117		1.910	-2.212	-1.158	
LiC5	Li-H	1.610	0.989	0.012	0.013	1380	9	0.007		0.261	-0.002	-0.007	
	H-H	2.003	0.077			509			-4.14	0.087	0.002	0.028	0.261
	H-C	1.075	5.697	-0.721	-0.112	3225	-198	-0.058		1.975	2.295	1.162	
LiC6	Li-H	1.610	0.988	0.011	0.012	1379	8	0.006		0.261	-0.002	-0.006	
	H-H	1.984	0.076			506			-4.38	0.090	0.002	0.025	0.260
	H-C	1.074	5.680	-0.814	-0.125	3220	-223	-0.065		1.958	-2.270	-1.160	
LiN1	Li-H	1.671	0.729	-0.248	-0.253	1185	-186	-0.136		0.232	0.001	0.005	
	H-H	1.679	0.138			681			-18.07	0.221	-0.026	-0.119	0.306
	H-N	1.076	3.755	-0.985	-0.208	2604	-321	-0.110		2.109	-2.981	-1.413	
LiN2	Li-H	1.612	0.916	-0.061	-0.062	1328	-43	-0.031		0.258	0.001	0.004	
	H-H	1.520	0.194			809			-13.85	0.248	-0.052	-0.212	0.337
	H-N	1.042	3.414	-4.602	-0.574	2483	-1321	-0.347		2.017	-3.574	-1.772	
LiO	Li-H	1.704	0.629	-0.348	-0.356	1100	-271	-0.198		0.219	0.002	0.008	
	H-H	1.643	0.066			472			-20.20	0.241	-0.036	-0.148	0.250
	H-O	1.002	3.146	-5.409	-0.632	2373	-1540	-0.394		2.177	-4.416	-2.028	
NaC1	Na-H	1.908	0.710	-0.001	-0.002	1117	-1	-0.001		0.217	0.008	0.036	
	H-H	2.485	0.026			296			-0.80	0.039	0.003	0.079	0.193
	H-C	1.089	5.305	-0.051	-0.010	3112	-15	-0.005		1.925	-2.152	-1.118	
NaC2	Na-H	1.944	0.661	-0.050	-0.071	1078	-40	-0.036		0.202	0.011	0.053	

Table S2 – Continued

Molecule	Bond	R	k^a	Δk^a	$\Delta k^a/k^a$	ω_a	$\Delta\omega_a$	$\Delta\omega_a/\omega_a$	E	ρ_c	H_c	H_c/ρ_c	n
	H-H	2.427	0.036			348			-10.08	0.063	0.001	0.022	0.211
	H-C	1.091	5.180	-0.089	-0.017	3075	-26	-0.008		2.014	-2.272	-1.128	
NaC3	Na-H	1.944	0.663	-0.048	-0.068	1079	-39	-0.035		0.203	0.011	0.052	
	H-H	2.231	0.057			438			-9.40	0.084	0.001	0.008	0.240
	H-C	1.093	5.103	-0.154	-0.029	3052	-46	-0.015		2.077	-2.377	-1.144	
NaC4	Na-H	1.907	0.710	-0.001	-0.002	1117	-1	-0.001		0.214	0.010	0.046	
	H-H	1.767	0.132			667			-9.72	0.145	-0.007	-0.050	0.303
	H-C	1.092	4.643	-1.643	-0.261	2911	-477	-0.141		1.892	-2.185	-1.155	
NaC5	Na-H	1.905	0.734	0.023	0.032	1136	18	0.016		0.217	0.008	0.038	
	H-H	2.001	0.079			517			-4.47	0.090	0.001	0.015	0.262
	H-C	1.076	5.588	-0.830	-0.129	3194	-229	-0.067		1.969	-2.284	-1.160	
NaC6	Na-H	1.905	0.732	0.021	0.029	1134	16	0.014		0.217	0.008	0.039	
	H-H	1.980	0.077			509			-4.75	0.093	0.001	0.011	0.261
	H-C	1.075	5.545	-0.949	-0.146	3182	-261	-0.076		1.950	-2.258	-1.158	
NaN	Na-H	1.975	0.583	-0.128	-0.181	1013	-105	-0.094		0.191	0.012	0.062	
	H-H	1.599	0.138			681			-15.89	0.251	-0.040	-0.159	0.306
	H-N	1.085	3.211	-1.529	-0.323	2407	-518	-0.177		2.056	-2.831	-1.377	
NaO	Na-H	2.022	0.408	-0.303	-0.427	847	-271	-0.242		0.177	0.012	0.067	
	H-H	1.572	0.113			617			-17.47	0.258	-0.050	-0.193	0.290
	H-O	1.005	3.573	-4.982	-0.582	2529	-1384	-0.354		2.151	-4.338	-2.017	
BeC1	Be-H	1.336	2.332	-0.027	-0.011	2090	-12	-0.006		0.653	-0.325	-0.498	
	H-H	2.047	0.044			387			-1.98	0.065	0.005	0.082	0.223
	H-C	1.070	6.031	-0.255	-0.041	3318	-70	-0.021		1.990	-2.326	-1.168	
BeC2	Be-H	1.335	2.359	0.000	0.000	2102	0	0.000		0.661	-0.334	-0.506	
	H-H	2.210	0.034			336			-1.03	0.046	0.005	0.118	0.208
	H-C	1.065	6.288	-0.130	-0.020	3388	-35	-0.010		2.011	-2.353	-1.170	
BeC3	Be-H	1.335	2.357	-0.002	-0.001	2101	-1	0.000		0.661	-0.334	-0.506	
	H-H	2.198	0.031			322			-1.06	0.047	0.005	0.117	0.202
	H-C	1.063	6.341	-0.153	-0.024	3402	-41	-0.012		1.998	-2.335	-1.169	
BeN1	Be-H	1.360	2.071	-0.288	-0.122	1969	-133	-0.063		0.586	-0.260	-0.445	
	H-H	1.549	0.223			867			-10.66	0.205	-0.030	-0.148	0.350
	H-N	1.045	5.150	-1.626	-0.240	3049	-449	-0.128		2.133	-3.502	-1.641	

Table S2 – Continued

Molecule	Bond	R	k^a	Δk^a	$\Delta k^a/k^a$	ω_a	$\Delta\omega_a$	$\Delta\omega_a/\omega_a$	E	ρ_c	H_c	H_c/ρ_c	n
BeN2	Be-H	1.388	1.743	-0.616	-0.261	1807	-295	-0.140		0.504	-0.190	-0.376	
	H-H	1.147	0.374			1123			-26.76	0.563	-0.318	-0.565	0.404
	H-N	1.165	1.194	-4.698	-0.797	1468	-1793	-0.550		1.579	-2.164	-1.370	
BeN3	Be-H	1.371	1.958	-0.401	-0.170	1915	-187	-0.089		0.554	-0.232	-0.418	
	H-H	1.349	0.305			1014			-16.22	0.339	-0.113	-0.333	0.382
	H-N	1.096	3.020	-2.809	-0.482	2335	-909	-0.280		1.898	-2.949	-1.554	
BeN4	Be-H	1.336	2.306	-0.053	-0.022	2078	-24	-0.011		0.647	-0.318	-0.492	
	H-H	1.801	0.082			525			-1.98	0.105	0.000	-0.001	0.265
	H-N	1.004	7.252	-0.764	-0.095	3618	-186	-0.049		2.272	-4.109	-1.809	
BeO1	Be-H	1.375	1.883	-0.476	-0.202	1878	-224	-0.107		0.536	-0.214	-0.399	
	H-H	1.237	0.406			1169			-21.18	0.431	-0.198	-0.459	0.414
	H-O	1.048	2.797	-4.740	-0.629	2238	-1435	-0.391		1.839	-3.655	-1.987	
BeO2	Be-H	1.382	1.761	-0.598	-0.253	1816	-286	-0.136		0.520	-0.200	-0.385	
	H-H	1.170	0.409			1173			-24.45	0.522	-0.281	-0.539	0.414
	H-O	1.076	1.775	-5.551	-0.758	1783	-1839	-0.508		1.721	-3.202	-1.860	
BeO3	Be-H	1.394	1.536	-0.823	-0.349	1696	-406	-0.193		0.497	-0.181	-0.365	
	H-H	1.091	0.351			1087			-29.61	0.653	-0.419	-0.641	0.397
	H-O	1.119	0.756	-6.418	-0.895	1163	-2421	-0.676		1.538	-2.457	-1.597	
BeO4	Be-H	1.405	1.574	-0.669	-0.298	1716	-334	-0.163		0.482	-0.168	-0.348	
	H-H	1.144	0.411			1177			-30.69	0.562	-0.323	-0.575	0.415
	H-O	1.089	1.448	-6.089	-0.808	1610	-2063	-0.562		1.635	-2.914	-1.782	
BeF	Be-H	1.342	2.188	-0.171	-0.072	2024	-78	-0.037		0.640	-0.310	-0.485	
	H-H	1.642	0.117			627			-4.08	0.163	-0.015	-0.091	0.293
	H-F	0.929	8.376	-1.343	-0.138	3854	-297	-0.072		2.448	-5.762	-2.354	
BeCl	Be-H	1.339	2.288	-0.071	-0.030	2070	-32	-0.015		0.649	-0.322	-0.495	
	H-H	1.834	0.067			474			-2.44	0.112	0.001	0.007	0.251
	H-Cl	1.290	4.653	-0.484	-0.094	2839	-144	-0.048		1.660	-1.663	-1.002	
BC1	B-H	1.233	2.881			2301				1.027	-1.018	-0.991	
	H-H	2.192	0.107			601				0.073	0.007	0.094	0.286
	H-C	1.089	5.301			3111				1.936	-2.173	-1.122	
BC2	B-H	1.240	2.755			2250				1.009	-0.991	-0.982	
	H-H	2.362	0.071			489				0.060	0.007	0.124	0.255

Table S2 – Continued

Molecule	Bond	R	k^a	Δk^a	$\Delta k^a/k^a$	ω_a	$\Delta\omega_a$	$\Delta\omega_a/\omega_a$	E	ρ_c	H_c	H_c/ρ_c	n
	H-C	1.090	5.267			3101				1.932	-2.170	-1.123	
BC3	B-H	1.237	2.773	-0.015	-0.005	2258	-6	-0.003		1.007	-0.986	-0.979	
	H-H	2.093	0.015			224			-19.83	-	-	-	0.165
	H-C	1.092	5.026	-1.260	-0.200	3029	-359	-0.106		1.896	-2.231	-1.177	
BC4	B-H	1.238	2.803	0.015	0.005	2270	6	0.003		1.004	-0.985	-0.981	
	H-H	2.533	0.016			231			-2.88	-	-	-	0.168
	H-C	1.090	5.304	-0.052	-0.010	3112	-15	-0.005		1.934	-2.154	-1.114	
BN1	B-H	1.241	2.717			2235				0.998	-0.974	-0.976	
	H-H	1.995	0.119			633				0.098	0.006	0.062	0.294
	H-N	1.015	6.729			3485				2.329	-3.465	-1.488	
BN2	B-H	1.257	2.423			2110				0.927	-0.865	-0.932	
	H-H	1.416	0.268			949				0.332	-0.086	-0.260	0.369
	H-N	1.069	3.225			2413				2.003	-3.116	-1.556	
BN3	B-H	1.244	2.669			2215				0.992	-0.966	-0.973	
	H-H	2.020	0.106			598				0.097	0.006	0.062	0.285
	H-N	1.016	6.715			3482				2.327	-3.428	-1.473	
BN4	B-H	1.265	2.211			2016				0.908	-0.836	-0.921	
	H-H	1.392	0.199			819				0.374	-0.112	-0.299	0.339
	H-N	1.083	2.398			2081				1.925	-2.928	-1.521	
BN5	B-H	1.240	2.766	-0.022	-0.008	2255	-9	-0.004		0.995	-0.969	-0.974	
	H-H	2.051	0.044			386			-8.42	0.089	0.004	0.047	0.223
	H-N	1.022	6.444	-0.548	-0.078	3411	-142	-0.040		2.276	-3.405	-1.496	
BO1	B-H	1.248	2.596			2184				0.972	-0.936	-0.962	
	H-H	1.774	0.142			691				0.148	-0.003	-0.023	0.309
	H-O	0.971	7.186			3587				2.442	-5.010	-2.052	
BO2	B-H	1.249	2.577			2177				0.973	-0.936	-0.963	
	H-H	1.796	0.125			648				0.146	-0.003	-0.022	0.298
	H-O	0.968	7.437			3649				2.460	-5.042	-2.049	
BS1	B-H	1.236	2.823			2278				1.014	-0.997	-0.984	
	H-H	1.929	0.111			610				0.113	0.004	0.037	0.288
	H-S	1.347	4.008			2639				1.484	-1.452	-0.978	
BS2	B-H	1.242	2.704			2229				0.997	-0.973	-0.976	

Table S2 – Continued

Molecule	Bond	R	k^a	Δk^a	$\Delta k^a/k^a$	ω_a	$\Delta\omega_a$	$\Delta\omega_a/\omega_a$	E	ρ_c	H_c	H_c/ρ_c	n
	H-H	1.985	0.073			497				0.105	0.004	0.040	0.257
	H-S	1.347	3.924			2611				1.481	-1.449	-0.978	
BF	B-H	1.243	2.598	-0.190	-0.068	2186	-78	-0.034		0.966	-0.920	-0.953	
	H-H	1.534	0.064			466			-20.77	0.240	-0.046	-0.192	0.248
	H-F	0.958	5.867	-3.852	-0.396	3226	-925	-0.223		2.182	-5.052	-2.316	
SiN	Si-H	1.511	2.554	-0.296	-0.104	2111	-119	-0.053		0.718	-0.415	-0.578	
	H-H	1.600	0.175			769			-7.45	0.175	-0.020	-0.112	0.327
	H-N	1.038	5.516	-1.260	-0.186	3156	-342	-0.098		2.175	-3.576	-1.644	
SiO	Si-H	1.537	2.200	-0.650	-0.228	1959	-271	-0.122		0.662	-0.363	-0.549	
	H-H	1.288	0.320			1038			-17.36	0.391	-0.161	-0.413	0.387
	H-O	1.039	3.003	-4.534	-0.602	2319	-1354	-0.369		1.894	-3.822	-2.018	
SiF	Si-H	1.492	2.729	-0.121	-0.042	2182	-48	-0.022		0.783	-0.480	-0.614	
	H-H	1.828	0.056			435			-1.51	0.099	0.000	0.004	0.238
	H-F	0.923	9.133	-0.586	-0.060	4025	-126	-0.030		2.510	-5.909	-2.354	
AlO1	Al-H	1.656	1.466	-0.019	-0.013	1600	-11	-0.007		0.443	-0.109	-0.246	
	H-H	1.674	0.141			688			-11.95	0.170	-0.017	-0.098	0.308
	H-O	0.974	6.961	-1.588	-0.186	3530	-382	-0.098		2.410	-4.964	-2.060	
AlO2	Al-H	1.667	1.360	-0.125	-0.084	1541	-70	-0.043		0.415	-0.092	-0.221	
	H-H	1.352	0.289			987			-23.34	0.347	-0.121	-0.350	0.376
	H-O	1.018	3.738	-4.612	-0.552	2587	-1279	-0.331		2.437	-3.943	-1.618	
AlF	Al-H	1.604	1.844	-0.377	-0.170	1795	-175	-0.089		0.529	-0.159	-0.300	
	H-H	2.278	0.031			324			-10.42	-	-	-	0.202
	H-F	0.930	8.462	-1.257	-0.129	3874	-277	-0.067		2.429	-5.842	-2.405	
GaF	Ga-H	1.575	2.082	-0.194	-0.085	1886	-86	-0.044		0.778	-0.409	-0.525	
	H-H	2.484	0.024			282			-6.25	-	-	-	0.188
	H-F	0.925	9.139	-0.580	-0.060	4026	-125	-0.030		2.486	-5.918	-2.380	
CF	C-H	1.089	5.196	-0.160	-0.030	3080	-47	-0.015		1.907	-2.148	-1.126	
	H-H	2.213	0.013			213			-1.79	-	-	-	0.159
	H-F	0.922	9.394	-0.325	-0.033	4082	-69	-0.017		2.521	-5.956	-2.363	
AB1	B-H	1.217	3.150	-0.179	-0.054	2406	-68	-0.027		1.107	-1.141	-1.030	
	H-H	2.003	0.029			313			-16.80	-	-	-	0.199

Table S2 – Continued

Molecule	Bond	R	k^a	Δk^a	$\Delta k^a/k^a$	ω_a	$\Delta\omega_a$	$\Delta\omega_a/\omega_a$	E	ρ_c	H_c	H_c/ρ_c	n
	H-N	1.013	7.044	0.005	0.001	3566	1	0.000		2.288	-3.631	-1.587	
AB2	B-H2	1.215	3.204	-0.125	-0.038	2427	-47	-0.019		1.112	-1.149	-1.033	
	B-H3	1.220	3.072	-0.257	-0.077	2376	-98	-0.040		1.096	-1.122	-1.024	
	H1-H2	1.916	0.046			394			-32.77	0.118	0.002	0.018	0.226
	H1-H3	2.181	0.028			309				-	-	-	
	H1-N	1.022	6.469	-0.570	-0.081	3417	-148	-0.042		2.299	-3.627	-1.577	
AB3	B-H2	1.220	3.064	-0.265	-0.080	2373	-101	-0.041		1.092	-1.117	-1.022	
	H1-H2	2.069	0.061			452			-48.05	0.097	0.005	0.056	0.244
	H1-N	1.020	6.635	-0.404	-0.057	3461	-104	-0.029		2.313	-3.628	-1.568	
AB4	B-H	1.216	3.219	-0.110	-0.033	2432	-42	-0.017		1.116	-1.156	-1.036	
	H-H	2.153	0.039			363			-8.64	-	-	-	0.216
	H-N	1.051	5.200	0.460	0.097	3064	139	0.048		2.261	-3.161	-1.398	
AB5	B-H	1.219	3.146	-0.183	-0.055	2405	-69	-0.028		1.101	-1.133	-1.028	
	H-H	1.990	0.038			356			-9.51	0.096	0.005	0.051	0.214
	H-O	0.966	7.791	-0.764	-0.089	3735	-178	-0.045		2.457	-5.066	-2.062	
AB6	B-H	1.216	3.210	-0.119	-0.036	2429	-45	-0.018		1.116	-1.157	-1.036	
	H-H	2.488	0.014			220			-10.46	-	-	-	0.162
	H-N	1.015	6.856	-0.136	-0.019	3518	-35	-0.010		2.315	-3.433	-1.483	
AB7	B-H	1.225	3.017	-0.312	-0.094	2355	-119	-0.048		1.066	-1.076	-1.009	
	H-H	1.582	0.114			619			-10.48	0.208	-0.028	-0.135	0.291
	H-F	0.941	7.091	-2.628	-0.270	3546	-605	-0.146		2.339	-5.487	-2.345	
FeN1	Fe-H	1.536	1.942	-0.023	-0.012	1825	-11	-0.006		0.802	-0.432	-0.539	
	H-H	1.967	0.08			518			-3.81	0.089	0.003	0.032	0.263
	H-N	1.015	6.761	-0.231	-0.033	3494	-59	-0.017		2.310	-3.406	-1.474	
FeN2	Fe-H	1.537	1.939	-0.026	-0.013	1823	-13	-0.007		0.804	-0.433	-0.539	
	H-H	2.198	0.033			335			-4.41	0.067	0.006	0.095	0.206
	H-N	1.015	6.789	-0.203	-0.029	3501	-52	-0.015		2.310	-3.430	-1.485	
FeO1	Fe-H	1.54	1.89	-0.075	-0.038	1800	-36	-0.020		0.788	-0.417	-0.529	
	H-H	1.701	0.131			664			-5.98	0.150	-0.011	-0.072	0.302
	H-O	0.968	7.475	-1.080	-0.126	3658	-255	-0.065		2.436	-5.014	-2.058	
FeO2	Fe-H	1.537	1.93	-0.035	-0.018	1819	-17	-0.009		0.806	-0.435	-0.539	

Table S2 – Continued

Molecule	Bond	R	k^a	Δk^a	$\Delta k^a/k^a$	ω_a	$\Delta\omega_a$	$\Delta\omega_a/\omega_a$	E	ρ_c	H_c	H_c/ρ_c	n
	H-H	2.037	0.02			262			-5.99	0.092	0.003	0.038	0.179
	H-O	0.967	7.713	-0.842	-0.098	3716	-197	-0.050		2.447	-5.048	-2.063	
FeF1	Fe-H	1.545	1.827	-0.138	-0.070	1770	-66	-0.036		0.769	-0.395	-0.514	
	H-H	1.451	0.254			924			-10.96	0.263	-0.065	-0.248	0.363
	H-F	0.948	6.343	-3.376	-0.347	3354	-798	-0.192		2.281	-5.241	-2.298	
FeF2	Fe-H	1.537	1.734	-0.231	-0.118	1724	-112	-0.061		0.804	-0.431	-0.536	
	H-H	1.802	0.008			161			-11.14	0.152	-0.011	-0.074	0.139
	H-F	0.942	7.16	-2.559	-0.263	3563	-589	-0.142		2.327	-5.448	-2.341	
KrO	Kr-H	1.769	0.845	-0.188	-0.182	1200	-127	-0.096		0.696	-0.307	-0.441	
	H-H	1.866	0.063			462			-3.10	0.111	-0.004	-0.032	0.246
	H-O	0.965	7.743	-0.812	-0.095	3723	-190	-0.049		2.469	-5.044	-2.043	
BeAr	Be-H	1.359	2.011	-0.348	-0.148	1940	-162	-0.077		0.592	-0.273	-0.461	
	H-H	1.322	0.169			755			-8.12	0.368	-0.135	-0.368	0.324
	H-Ar	1.373	1.807	-1.346	-0.427	1766	-567	-0.243		1.341	-1.239	-0.924	
BeKr	Be-H	1.346	2.195	-0.164	-0.070	2027	-75	-0.036		0.632	-0.308	-0.487	
	H-H	1.674	0.082			527			-3.65	0.167	-0.010	-0.061	0.265
	H-Kr	1.485	2.585	-0.289	-0.101	2099	-114	-0.052		1.273	-1.044	-0.820	
Ar1	C-H	1.083	5.564			3187				1.975	-2.249	-1.139	
	H-H	2.472	0.271			955				-	-	-	0.370
Ar2	C-H	1.083	5.569			3188				1.975	-2.251	-1.139	
	H-H	2.464	0.273			959				-	-	-	0.370
Ar3	C-H	1.080	5.651			3212				1.989	-2.278	-1.145	
	H-H	1.998	0.296			999				0.090	0.013	0.141	0.379
H2	H-H	0.743	5.835	0.000	0.000	4433	0	0.000	-107.34	1.820	-2.047	-1.125	1.000
H2P	H-H	1.105	1.263	0.000	0.000	2062	0	0.000	-68.57	0.596	-0.499	-0.837	0.500
H3P	H-H	0.878	2.737			3036			-107.42	1.611	-1.537	-0.954	0.710
H4P	H1-H3	1.259	0.177			773			-12.00	0.440	-0.234	-0.532	0.205
	H1-H2	1.000	0.806			1648				1.176	-0.962	-0.818	0.408
	H2-H2	0.829	3.479			3423				1.531	-1.475	-0.963	0.791
H5P(D_{2d})	H1-H2	1.134	0.219			858			-29.86	0.821	-0.548	-0.667	0.226

Table S2 – Continued

Molecule	Bond	R	k^a	Δk^a	$\Delta k^a/k^a$	ω_a	$\Delta\omega_a$	$\Delta\omega_a/\omega_a$	E	ρ_c	H_c	H_c/ρ_c	n
	H2-H2	0.787	3.683			3522				1.655	-1.721	-1.040	0.812
H6P	H1-H1	1.017	1.079			1906				0.832	-0.707	-0.850	0.466
	H1-H3	1.252	0.297			1000			-21.34	0.624	-0.288	-0.461	0.260
	H3-H3	0.794	4.235			3777				1.565	-1.639	-1.047	0.865
H7P	H1-H2	0.881	1.181			1994				1.456	-1.371	-0.942	0.485
	H2-H2	0.983	1.119			1941				-	-	-	0.473
	H2-H3	1.556	0.101			584			-8.18	0.280	-0.054	-0.194	0.159
	H3-H3	0.758	5.130			4157				1.750	-1.917	-1.095	0.943
H9P	H1-H1	0.905	1.558			2291				1.509	-1.374	-0.910	0.550
	H1-H3	1.675	0.113			616			-5.98	0.211	-0.022	-0.103	0.168
	H3-H3	0.754	5.376			4255				1.768	-1.950	-1.103	0.963
LiH	Li-H	1.615	0.977			1371				0.260	-0.002	-0.008	
BeH	Be-H	1.335	2.359			2102				0.665	-0.339	-0.509	
BH4	B-H	1.239	2.788			2264				1.003	-0.984	-0.981	
CH4	C-H	1.088	5.356			3127				1.915	-2.165	-1.131	
NH3	N-H	1.011	6.992			3553				2.336	-3.365	-1.441	
NH4	N-H	1.022	6.776			3498				2.292	-3.749	-1.636	
H2O	O-H	0.957	8.555			3913				2.536	-5.153	-2.032	
H3O	O-H	0.975	7.537			3673				2.325	-5.013	-2.156	
HF	F-H	0.918	9.719			4151				2.557	-5.990	-2.342	
NaH	Na-H	1.908	0.711			1118				0.216	0.010	0.044	
AlH3	Al-H	1.583	2.221			1970				0.558	-0.177	-0.316	
AlH4	Al-H	1.646	1.485			1611				0.461	-0.120	-0.260	
GaH3	Ga-H	1.562	2.276			1972				0.806	-0.439	-0.545	
SiH4	Si-H	1.483	2.850			2230				0.813	-0.512	-0.630	
HCl	Cl-H	1.280	5.137			2983				1.700	-1.714	-1.008	
HOCl2	O-H	0.982	7.174			3584				2.328	-4.992	-2.144	
HOCl	O-H	0.979	7.326			3622				2.326	-4.991	-2.146	
KrH	Kr-H	1.720	1.033			1327				0.775	-0.391	-0.504	
ArF	Ar-H	1.332	3.153			2333				1.513	-1.423	-0.940	
KrF	Kr-H	1.477	2.874			2213				1.296	-1.069	-0.824	

Table S2 – Continued

Molecule	Bond	R	k^a	Δk^a	$\Delta k^a/k^a$	ω_a	$\Delta\omega_a$	$\Delta\omega_a/\omega_a$	E	ρ_c	H_c	H_c/ρ_c	n
C2H2	C-H	1.062	6.418			3423				2.020	-2.375	-1.176	
C2HF	C-H	1.060	6.494			3443				2.008	-2.360	-1.175	
HCN	C-H	1.066	6.286			3388				-	-	-	
HNC	N-H	0.995	8.016			3804				2.335	-4.165	-1.784	
CHF	C-H	1.090	5.269			3101				1.994	-2.258	-1.132	
CHF2	C-H	1.091	5.257			3098				2.067	-2.355	-1.139	
CHO	O-H	0.956	8.549			3912				2.564	-5.198	-2.027	
CFO	O-H	0.961	8.350			3866				2.494	-5.144	-2.062	
HNO	N-H	1.057	4.740			2925				2.220	-2.849	-1.283	
H2NO	N-H	1.042	5.829			3244				2.226	-3.686	-1.656	
BeBe	Be-H	1.344	2.243			2050				0.653	-0.328	-0.502	
FeH	Fe-H	1.535	1.965			1836				0.810	-0.442	-0.545	

[a] Bond length values R in Å, force constant k^a in mdyn/Å, vibrational frequency ω_a in cm^{-1}, energy E in kcal/mol, electron density at critical points ρ_c in e/Å^3, energy density at critical points H_c in Hartree/Å^3.
